# The Physiology and Pathology of the Cerebral Circulation

**Published:** 1898-06

**Authors:** 


**The Physiology and Pathology of the Cerebral Circulation.** By LEONARD HILL, M.B. Pp. xvi., 208. London: J. & A. Churchill. 1896.

In these pages we have the records of an extensive experimental research in the above branch of physiology, and also a summary of the previously recorded work of others. The author describes with lucidity the numerous and varied experiments he has made in the attempt to shed light upon the problem of the cerebral circulation. That he has exercised great care and ingenuity in devising and performing these experiments, many of which were delicate and complicated, is obvious, and the conclusions he has drawn from them appear to be fully justified. The mutability of physiological and pathological doctrine has been so frequently illustrated in the past that we are compelled to approach new theories and explanations of phenomena in these sciences with extreme caution. We anticipate however that future investigation will confirm many of the author's conclusions. It is impossible here to more than allude to one or two of Mr. Hill's researches and views.

In the section upon the cerebro-spinal fluid he points out the impossibility of maintaining that fluid in a state of higher pressure than that of the blood in the veins and sinuses of the meninges, for the fluid easily passes into these vessels when its pressure is greater. This fluid cannot therefore transmit increased pressure, except for a very brief period.

In no experiment has the author discovered evidence of nervous vaso-motor control of the intra-cranial vessels. In all his experiments, changes in the cerebral circulation appeared to passively follow the changes in the general arterial and venous pressures. It is a little hard to believe that the vessels in the cranium, peculiar as their situation is in a rigid cavity of unalterable size, differ so fundamentally from the extra-cranial vessels in this respect. The researches upon the effect of gravity on the circulation are very interesting. If the splanchnic vaso-motor constrictors be paralysed and the head then raised, syncope results. This is recovered from if the head be lowered. The blood in fact, under these circumstances, passively obeys gravity. Excessive inhalation of chloroform tends to paralyse the splanchnics. Hence the importance of keeping the head low in such anæsthesia.

In the sections on cerebral anæmia and cerebral compression it is shown that the culminating symptoms and the mode of death are the same in each case. Death results when the bulbar circulation ceases. Usually the respiratory centre is paralysed before the vaso-motor. Though the functions of the brain can be performed under any pressure from zero to 50 mm. Hg, yet if the cause which increases or decreases pressure also produces anæmia of the bulb to a considerable degree, the vital centres are paralysed.

We can unhesitatingly say that the volume before us, the outcome of a vast amount of experimental work, materially advances the study of the branch of physiology with which it deals, and merits the most careful attention of all scientific physicians and surgeons as well as of physiologists.

